# Pictorial essay: Distal colostography

**DOI:** 10.4103/0971-3026.63054

**Published:** 2010-05

**Authors:** Mukund D Rahalkar, Anand M Rahalkar, Dilip M Phadke

**Affiliations:** Department of Radiology, Sahyadri Hospital, Pune, India; 1 Department of Paediatrics, Sahyadri Hospital, Pune, India

**Keywords:** Imperforate anus, imaging of anorectal malformations, pouch colon

## Abstract

Distal colostography (DC), also called distal colography or loopography, is an important step in the reparative management of anorectal malformations (ARMs) with imperforate anus, Hirschsprung's disease (occasionally) and colonic atresia (rarely) in children and obstructive disorders of the distal colon (colitis with stricture, carcinoma or complicated diverticulosis) in adults. It serves to identify/confirm the type of ARM, presence/absence of fistulae, leakage from anastomoses, or patency of the distal colon. We present a pictorial essay of DC in a variety of cases.

## Introduction

Distal colostography (DC) is an important diagnostic investigation to delineate the altered anatomy of anorectal malformations and know the spectrum of associated fistulae between the blind rectum on the one hand and the bladder, urethra, perineum and vagina on the other. It remains a dependable test for a surgeon to plan surgical repair.

## Discussion

Anorectal malformations (ARMs) occur with an incidence of 1 in 5000[1] and their management is now well established, with immediate neonatal diverting colostomy in the high type of anomalies or anoplasty in the low type of anomalies.

**Figure 1:**
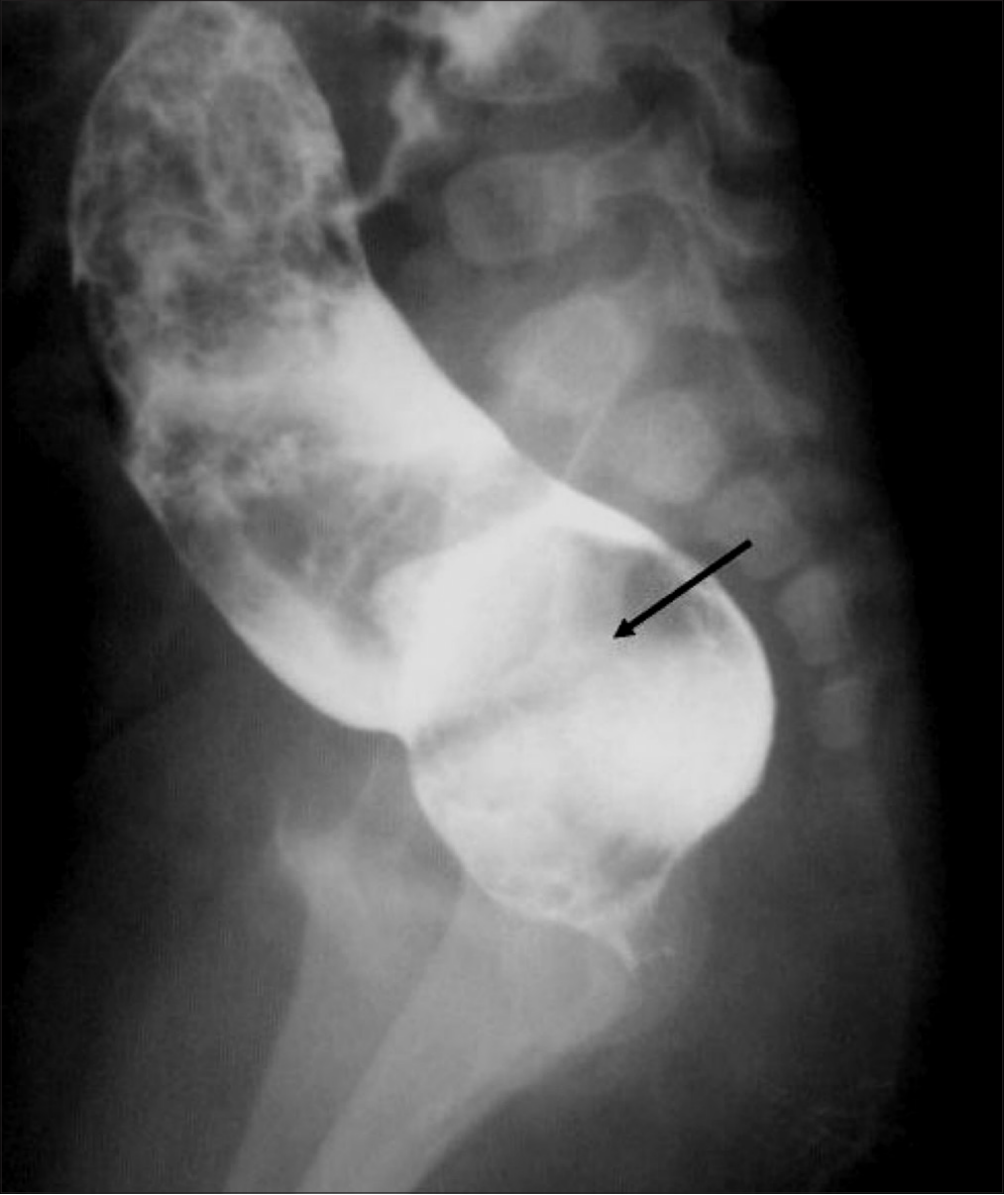
Blind end of rectum with faecal residue. The rectum is greatly distended and extending above the level of ischial tuberosities and so suggestive of an intermediate type of ARM. It is also significantly loaded with meconium / faecal residue (arrow marks rectum)

**Figure 2:**
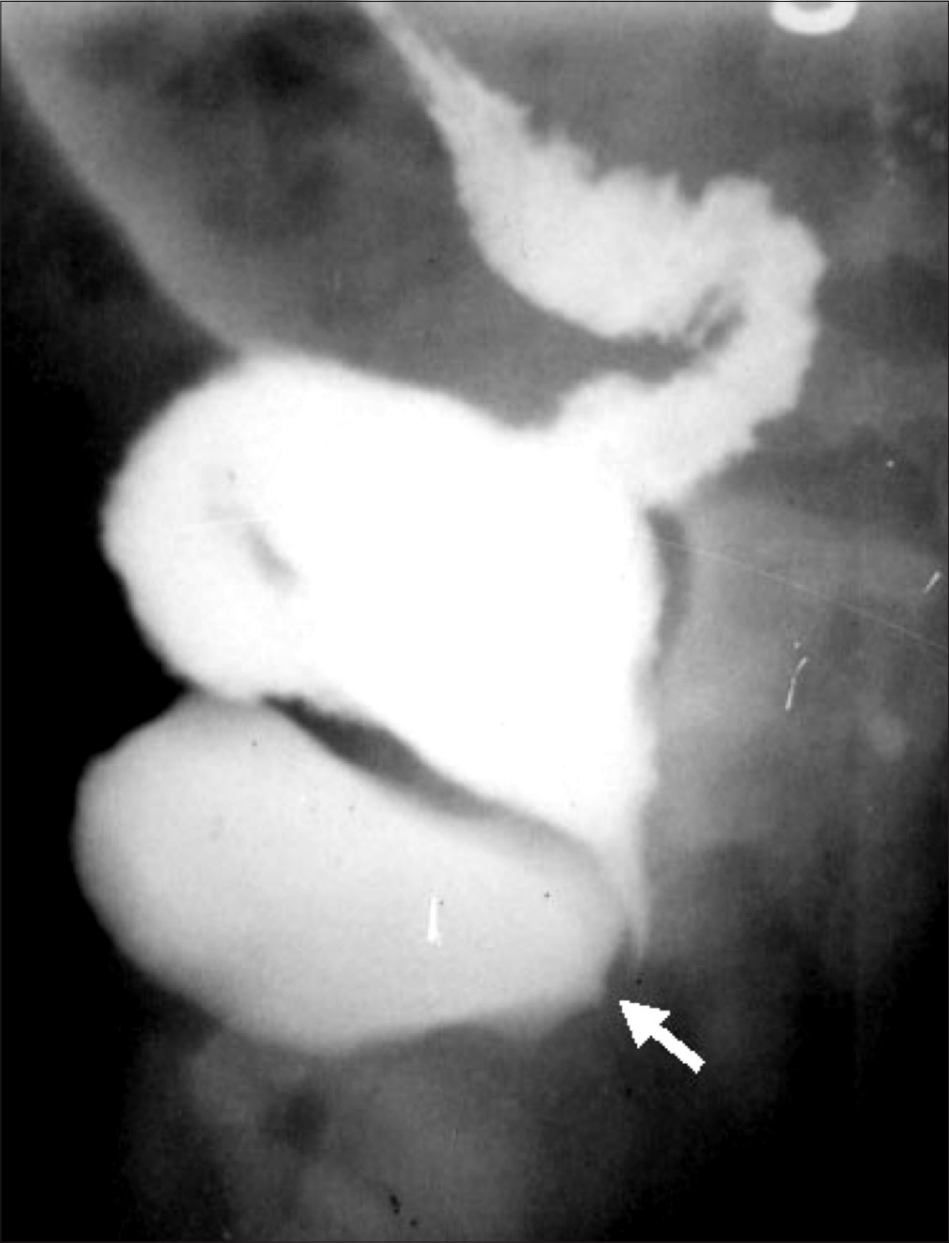
Recto–bladder neck fistula (arrow). The distal colostogram shows that the distal colon opens into the bladder neck and so this is a high type of ano-rectal malformation. (R: rectum and B: bladder)

**Figure 3:**
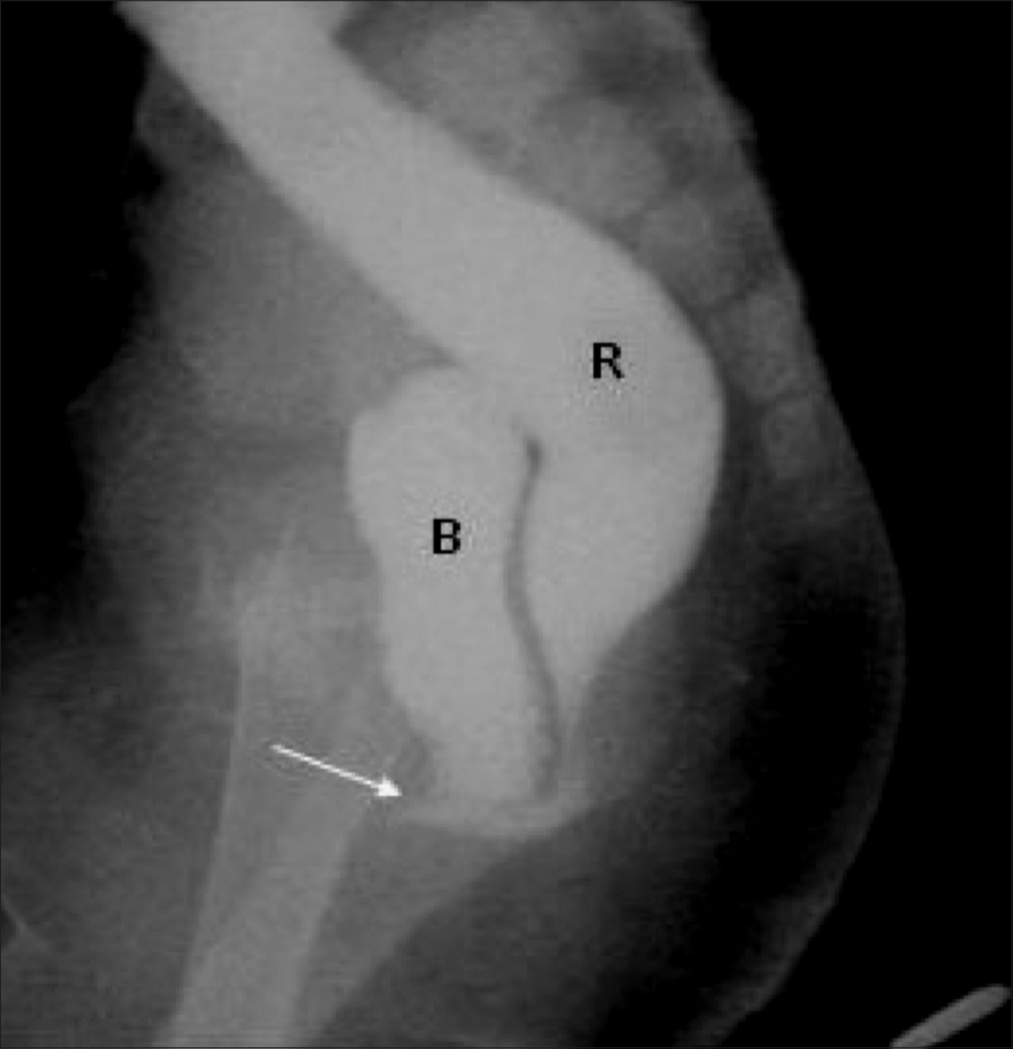
Recto-prostsatic urethral fistula. The DC shows that the distal colon is opening into the prostatic urethra. The contrast is refluxing proximally into bladder and also getting micturated. This is associated with a high type of ARM. (R: Rectum, B: bladder and arrow points to recto - prostatic urethral fistula)

**Figure 4 (A, B):**
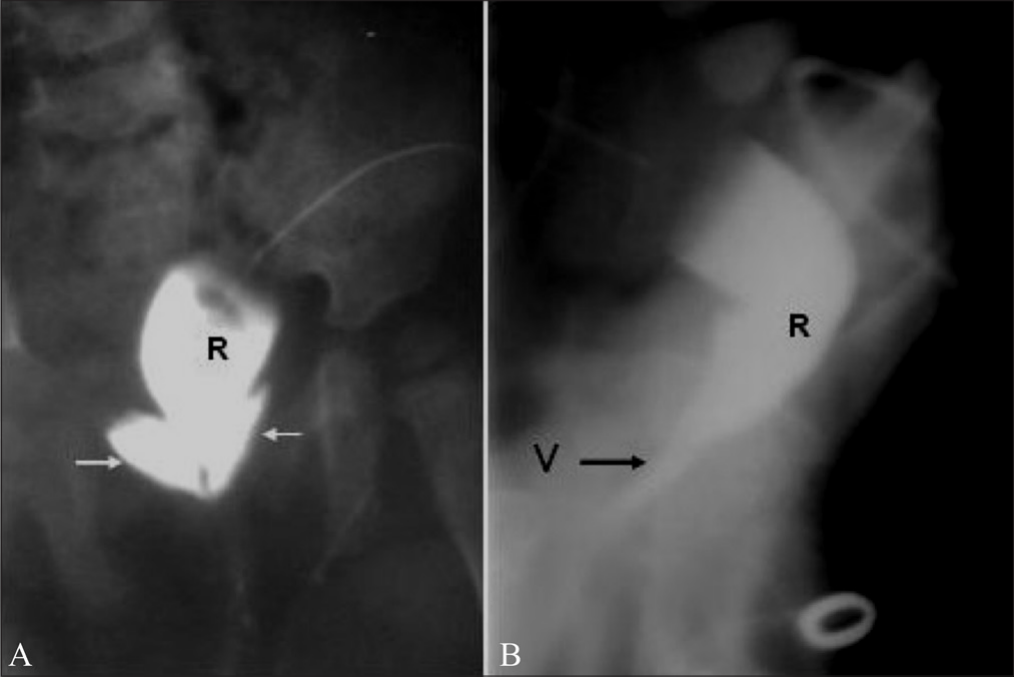
Recto-vaginal fistula. The DC reveals that the distal colon is opening into upper end of the vagina, making this a high type of ARM. Anteroposterior view shows opacification of the fornices. Arrows in 4A mark the vaginal fornices. (R: rectum, V: vagina)

**Figure 5:**
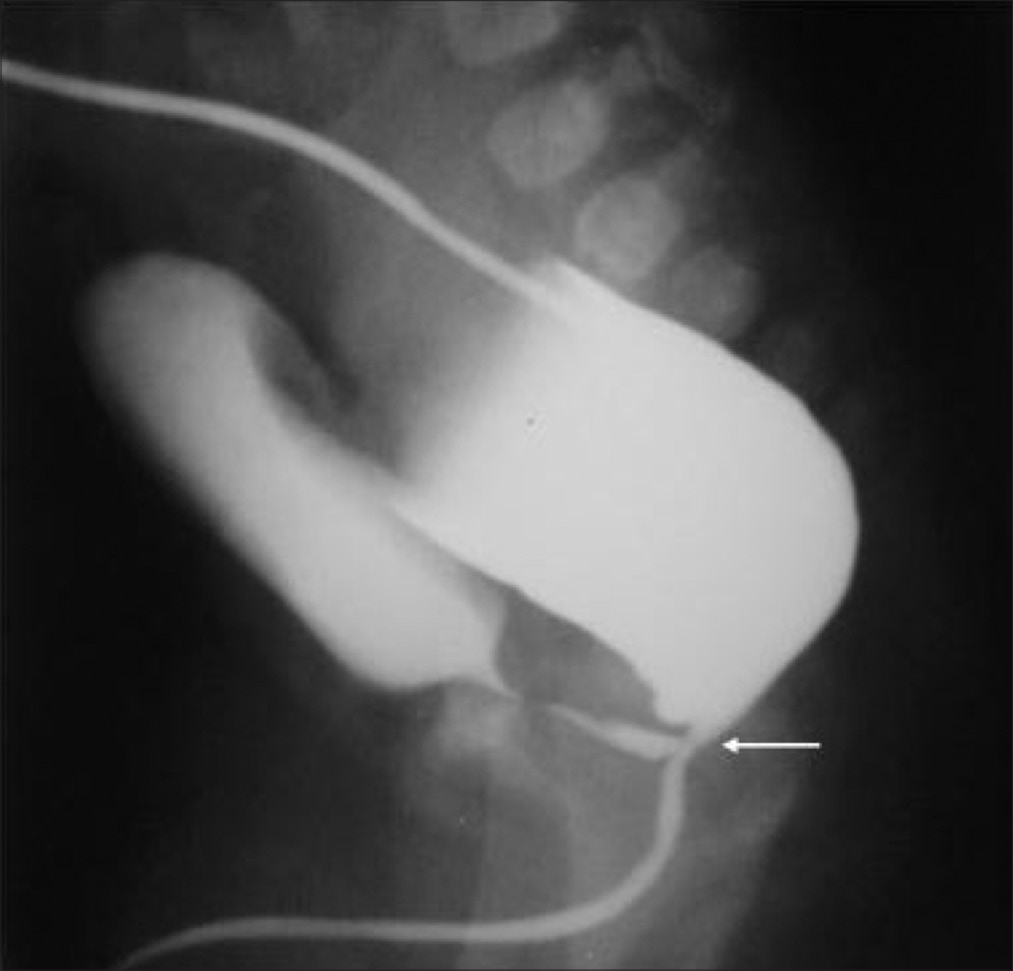
Recto- (bulbar) urethral fistula. The DC shows that the distal rectum is communicating with bulbar urethra and distal urethra is opacified. The urethra is kinked acutely at the site of fistula (marked by an arrow). This is an intermediate type of ARM

**Figure 6:**
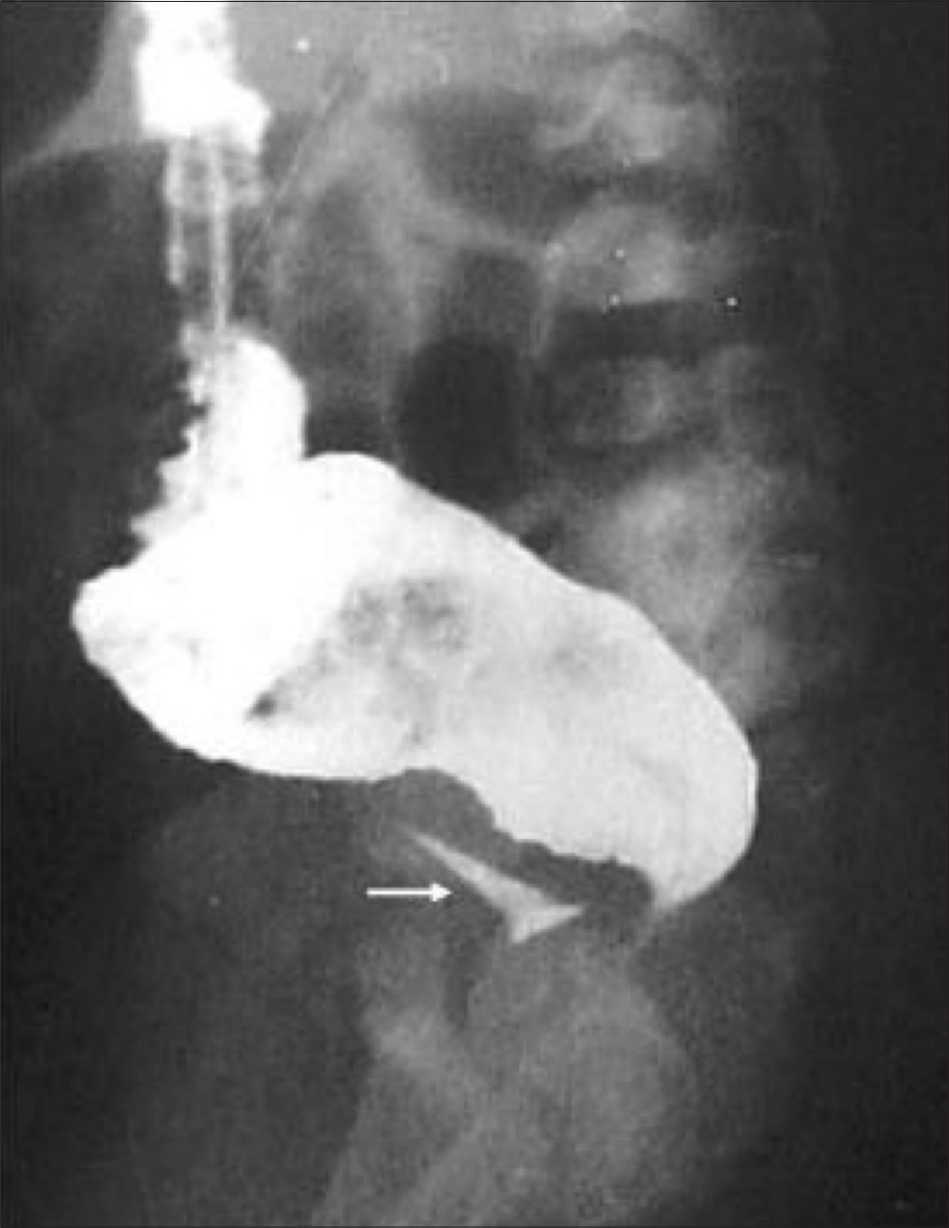
Recto-vaginal fistula. The rectum is opening into lower part of vagina (marked by an arrow) and so is associated with an intermediate or low type of ARM

**Figure 7:**
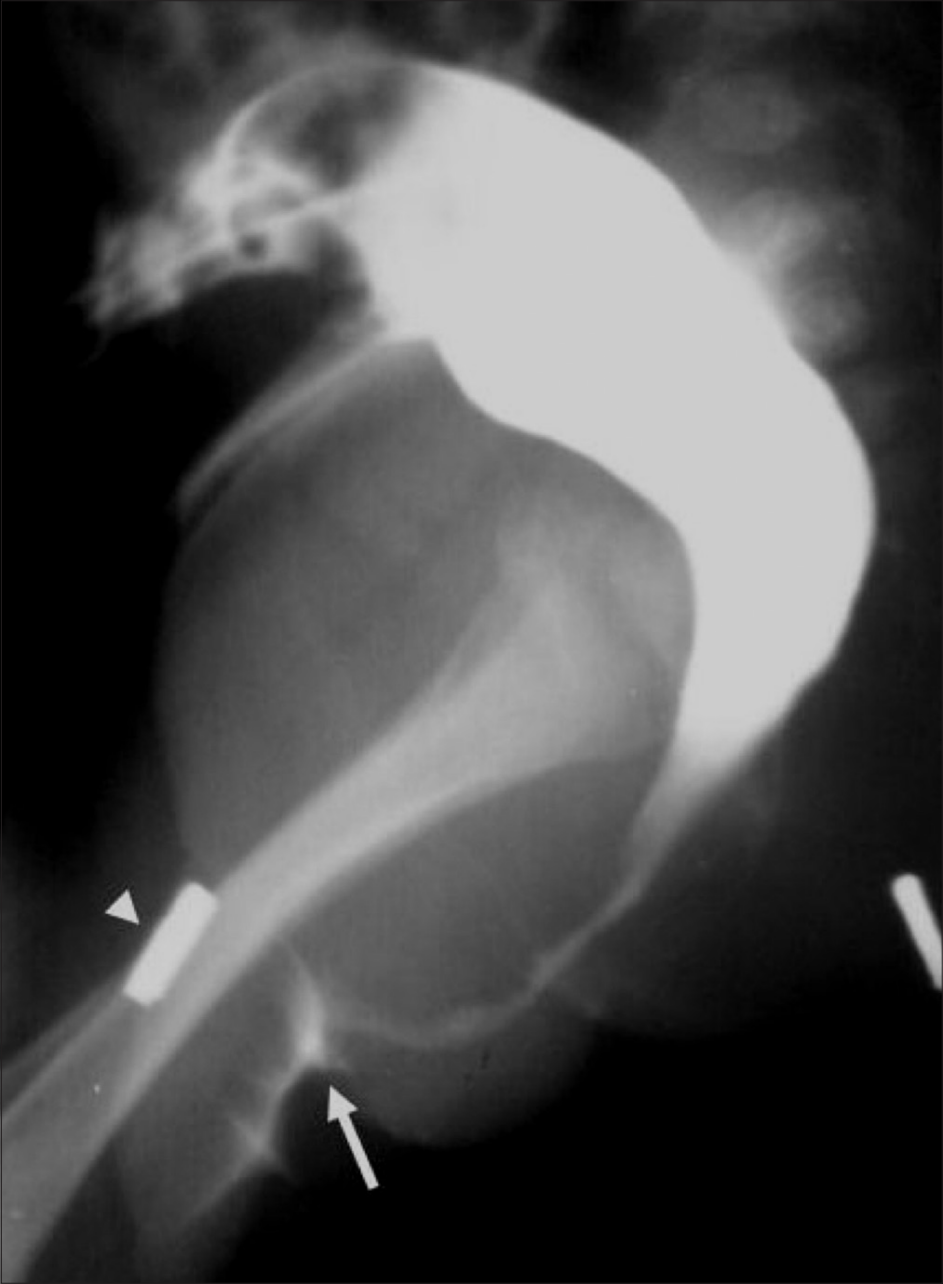
Recto-perineal fistula: The distal colon is opening at the base of a hypospadiac penis (marked by an arrow). A metallic marker was placed over the base of penis on dorsal aspect (marked by arrowhead). This is associated with a low type of ARM. (P: penis)

**Figure 8:**
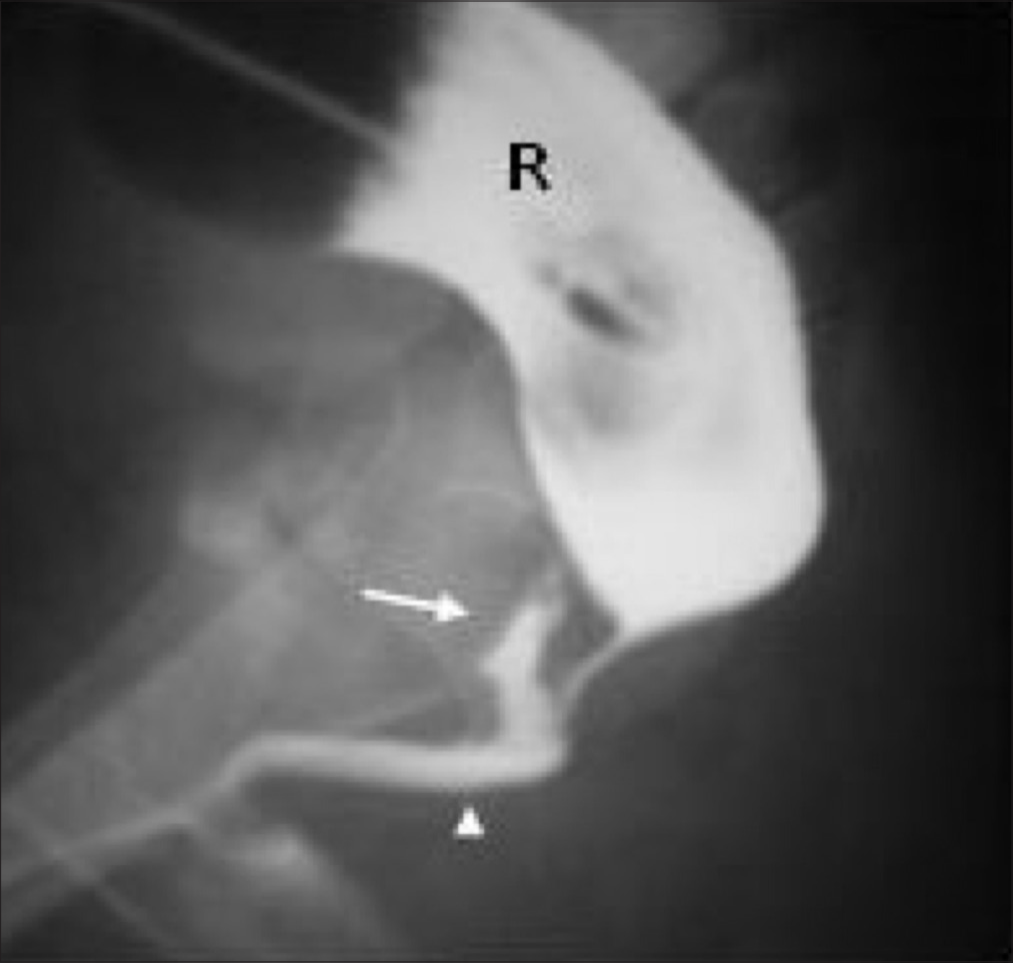
Cloaca in a female child. The DC shows rectum and uterus opening into a common chamber of cloaca marked by an arrow head. The contrast filled the common chamber and vaginal cavity (marked by an arrow), while it was not filing bladder retrogradely. The urethral opening was also noticed to be inside the cloaca

**Figure 9:**
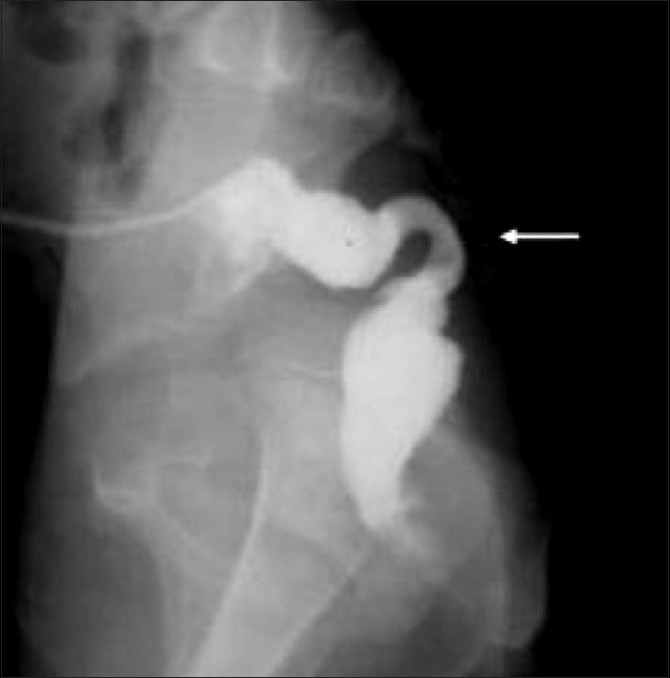
Pouch colon with cloaca. At surgery, the entire colon was absent except for a rectal pouch, into which the terminal ileum was opening. Hence an ileostomy was performed. Only a single opening of the cloaca was noted. The ileostomogram instead of a distal colostogram in this 2-year-old female child opacifies the common chamber of the cloaca (thick arrow) and shows the fistula (arrowhead) (P - pouch colon). The terminal ileum is seen as well (thin arrow)

About one month after colostomy or before the reparative surgery is planned, distal colostography (DC) is essential. It serves many purposes;[2] it helps the surgeon to:

- Find the degree of fecal impaction and ectasia of the blind end of the rectum [Figure 1]. Prior information about the distended rectum helps the surgeon to plan the rectal pull-through surgery.
- Judge the distance of the blind rectum from the marker placed at the expected site of the anus (pouch-to-perineum distance)
- Detect precisely the various types of rectal fistulae[3,4] [Figures 2–7], cloaca[5] [Figure 8] and pouch colon[6] [Figure 9].

According to Durham,[5] Keiller was the first to describe the DC technique of injecting barium sulphate to visualize the distal blind end. He advised washouts of the distal colon and removal of the accumulated meconium before injection of contrast. Later, along with others, Cremin[7,8] established the technique of DC in 1972. He insisted that the injection of contrast should be pressure-augmented. Gross[9] also stressed the value of the augmented pressure technique, where continued pressure is to be applied during injection to ensure that the fistula is opacified.

The technique followed by us is as follows:

1. A marker is placed over the anal dimple or expected position of the anus. Another marker is placed at the point where urine or fecal material is seen to be discharging.
2. After passing an indwelling catheter through the stoma leading to the distal colon, its balloon is inflated and it is pulled back during injection of the contrast to avoid any spillage. The distal blind end of the colon gets filled progressively and pressure is maintained till the contrast fills the fistulous tract.
3. Water-soluble contrast is used.
4. Images are obtained under fluoroscopy.
5. The colostogram is obtained in the lateral position, with the femora overlapping as perfectly as possible, to determine the level of the blind end of the rectum and identify the type of ARM.

In practice, DC is a very useful technique since it has a high specificity. Its sensitivity can be increased if proper care is taken to demonstrate the most distal end of the blind rectum and the fistula.[10]

### Hirschsprung's disease

**Figure 10:**
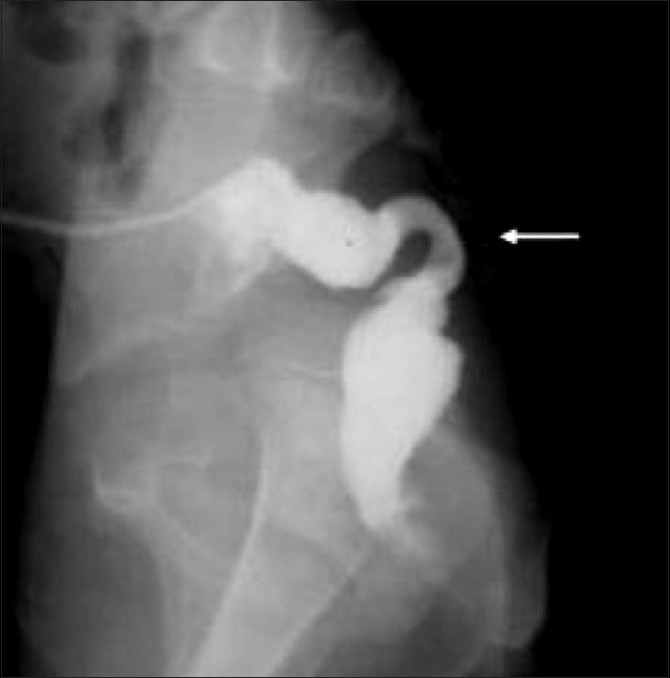
Colostomy for Hirschprung's disease. Some pediatric surgeons carry out colostomy in early management of Hirschprung's disease. DC in a child in whom colostomy was carried out for a significantly narrow aganglionic segment (marked by an arrow) confirmed the diagnosis. Note that the proximal colon looks small after defunctioning colostomy

Some surgeons perform a defunctioning colostomy for the management of the aganglionic colon. DC confirms the earlier diagnosis and helps in planning the further course of action [Figure 10].

### Recto sigmoid obstructive disorders

**Figure 11:**
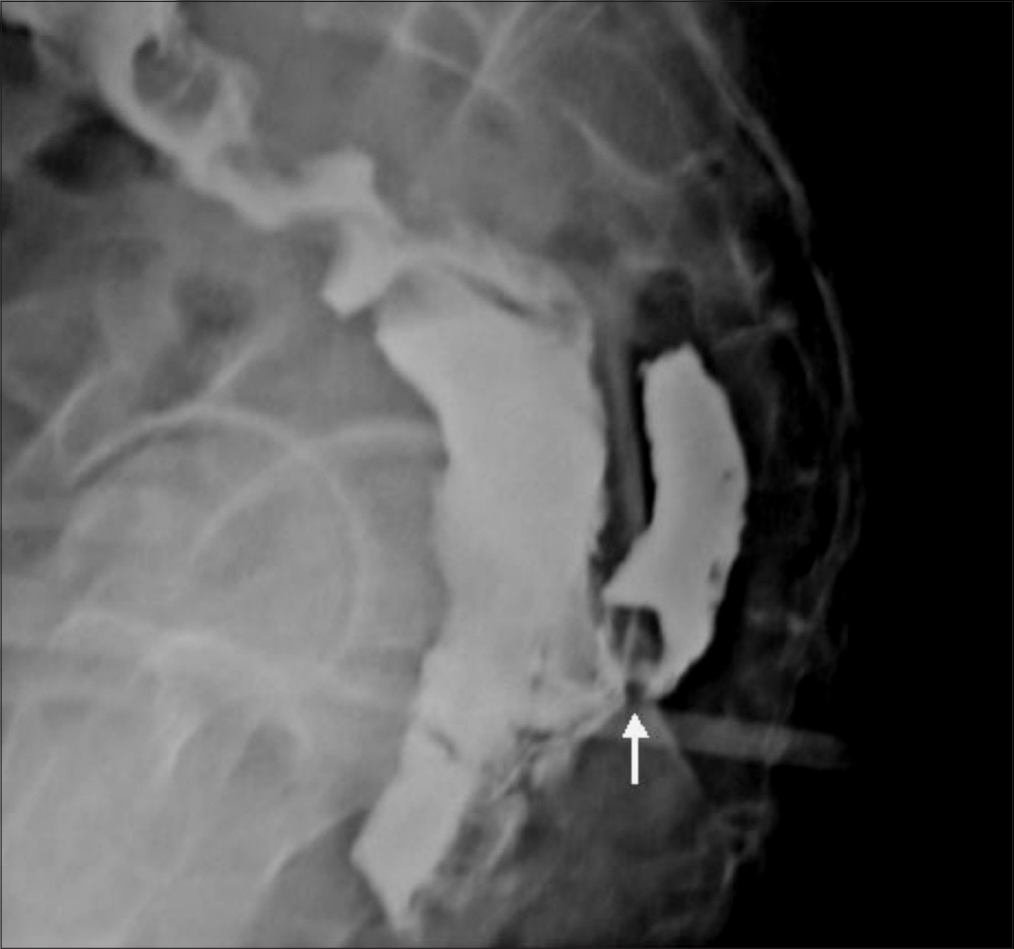
DC for checking the lumen of distal colon. : DC shows leakage of contrast from the site of anastomosis after resection of a malignant growth of recto sigmoid in an old person. Based on this observation the closure of colostomy was deferred for some more time

In dealing with strictures due to chronic colitis or complicated diverticulosis and malignant tumors of the rectosigmoid region a defunctioning colostomy and resection with anastomosis are undertaken. DC is useful to check for any leakage from the site of anastomosis before closure of the colostomy [Figure 11].
